# Antibody Mediated Rejection and T-cell Mediated Rejection Molecular Signatures Using Next-Generation Sequencing in Kidney Transplant Biopsies

**DOI:** 10.3389/ti.2024.13043

**Published:** 2024-07-10

**Authors:** Esteban Cortes Garcia, Alessia Giarraputo, Maud Racapé, Valentin Goutaudier, Cindy Ursule-Dufait, Pierre de la Grange, Lucie Adoux, Marc Raynaud, Clément Couderau, Fariza Mezine, Jessie Dagobert, Oriol Bestard, Francesc Moreso, Jean Villard, Fabian Halleck, Magali Giral, Sophie Brouard, Richard Danger, Pierre-Antoine Gourraud, Marion Rabant, Lionel Couzi, Moglie Le Quintrec, Nassim Kamar, Emmanuel Morelon, François Vrtovsnik, Jean-Luc Taupin, Renaud Snanoudj, Christophe Legendre, Dany Anglicheau, Klemens Budde, Carmen Lefaucheur, Alexandre Loupy, Olivier Aubert

**Affiliations:** ^1^ Université Paris Cité, INSERM U970, Paris Institute for Transplantation and Organ Regeneration, Paris, France; ^2^ GenoSplice, Paris, France; ^3^ Université Paris Cité, Centre National de la Recherche Scientifique (CNRS), INSERM, Institut Cochin, Paris, France; ^4^ Department of Nephrology and Kidney Transplantation, Vall d'Hebron Hospital Universitari, Vall d'Hebron Institut de Recerca (VHIR), Vall d'Hebron Barcelona Hospital Campus, Universitat Autònoma de Barcelona, Barcelona, Spain; ^5^ Transplantation Immunology Unit and National Reference Laboratory for Histocompatibility, Department of Diagnostic, Geneva University Hospitals, Geneva, Switzerland; ^6^ Department of Nephrology and Intensive Care, Charité-Universitätsmedizin Berlin, Corporate Member of Freie Universität Berlin and Humboldt-Universität zu Berlin, Berlin, Germany; ^7^ Nantes Université, INSERM, CRT2I-Center for Research in Transplantation and Translational Immunology, Nantes, France; ^8^ Nantes Université, Centre Hospitalier Universitaire de Nantes, Pôle Hospitalo-Universitaire 11: Santé Publique, Clinique des données, INSERM, Centre d’Investigation Clinique 1413, Nantes, France; ^9^ Department of Pathology, Necker-Enfants Malades Hospital, Assistance Publique - Hôpitaux de Paris, Université Paris Cité, Paris, France; ^10^ Centre Hospitalier Universitaire de Bordeaux, Service de Néphrologie, Transplantation, Dialyse et Aphérèses, Bordeaux, France; ^11^ Department of Nephrology Dialysis and Kidney Transplantation, Centre Hospitalier Universitaire de Montpellier, Montpellier, France; ^12^ Department of Nephrology and Organ Transplantation, Toulouse Rangueil University Hospital, INSERM UMR 1291, Toulouse Institute for Infectious and Inflammatory Diseases (Infinity), University Paul Sabatier, Toulouse, France; ^13^ Department of Transplantation, Nephrology and Clinical Immunology, Hospices Civils de Lyon, Lyon, France; ^14^ Department of Kidney Transplantation, Bichat Hospital, Assistance Publique—Hôpitaux de Paris (APHP), Paris, France; ^15^ Laboratory of Immunology and Histocompatibility, Hôpital Saint-Louis Assistance Publique - Hôpitaux de Paris, Paris, France; ^16^ Assistance Publique des Hôpitaux de Paris (AP-HP), Université Paris-Saclay, Hôpital de Bicêtre, Service de Néphrologie et Transplantation, Le Kremlin-Bicêtre, France; ^17^ Department of Kidney Transplantation, Necker Hospital, Assistance Publique—Hôpitaux de Paris, Paris, France; ^18^ Kidney Transplant Department, Saint-Louis Hospital, Assistance Publique—Hôpitaux de Paris, Paris, France

**Keywords:** next generation sequencing, RNA-seq experiment, kidney biopsies, molecular signature, allograft rejection, kidney transplantation

## Abstract

Recently, interest in transcriptomic assessment of kidney biopsies has been growing. This study investigates the use of NGS to identify gene expression changes and analyse the pathways involved in rejection. An Illumina bulk RNA sequencing on the polyadenylated RNA of 770 kidney biopsies was conducted. Differentially-expressed genes (DEGs) were determined for AMR and TCMR using DESeq2. Genes were segregated according to their previous descriptions in known panels (microarray or the Banff Human Organ Transplant (B-HOT) panel) to obtain NGS-specific genes. Pathway enrichment analysis was performed using the Reactome and Kyoto Encyclopaedia of Genes and Genomes (KEGG) public repositories. The differential gene expression using NGS analysis identified 6,141 and 8,478 transcripts associated with AMR and TCMR. While most of the genes identified were included in the microarray and the B-HOT panels, NGS analysis identified 603 (9.8%) and 1,186 (14%) new specific genes. Pathways analysis showed that the B-HOT panel was associated with the main immunological processes involved during AMR and TCMR. The microarrays specifically integrated metabolic functions and cell cycle progression processes. Novel NGS-specific based transcripts associated with AMR and TCMR were discovered, which might represent a novel source of targets for drug designing and repurposing.

## Graphical Abstract

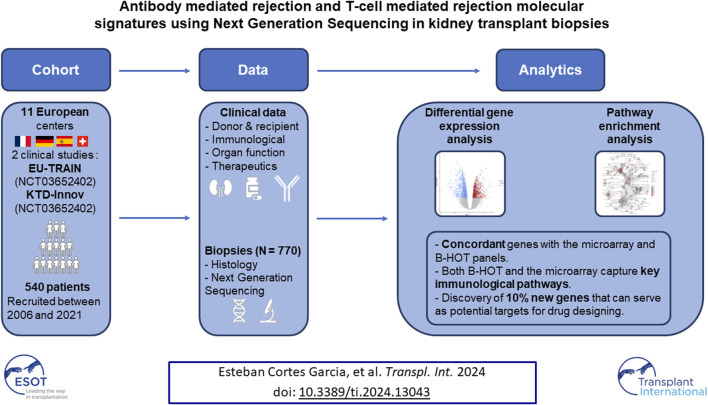

## Introduction

Long-term kidney allograft survival is mainly limited by the occurrence of rejections [1, 2]. To improve kidney injury detection, the biennial revision of the Banff classification emerged as the gold standard for the diagnosis of rejection during the past 3 decades [3, 4]. From histology assessment of kidney biopsies, combined with clinical and immunological parameters, the classification is now encompassing molecular and digital biomarkers to improve its sensitivity and provide new diagnostic tools for the clinicians. Recently, transcriptome analysis has shown its capacity to accurately detect injuries and the degree of activity in solid organ transplant biopsies [5]. Previous studies focusing on the implementation of microarrays paved the way for the molecular understanding of rejection and allowed the development of gene expression-based classifiers [6]. However, this technology suffers from its necessity to design probes, limiting the past studies to the coding transcriptome only.

While lacking protein-coding ability, long noncoding RNAs (lncRNAs) act as functional RNA molecules, regulating protein-coding gene expression through interactions with gene-regulatory proteins and microRNAs. Growing evidences in the literature showed the pivotal role played by lncRNAs in the establishment and maintenance of the immune response [7–9]. Therefore, they represent a complete novel source of biomarkers for the diagnosis of various cancers [10–12]. However, lncRNAs implication in the solid organ transplantation field remains poorly investigated. Combining the non-coding transcriptome on top of the coding might help our understanding of the pathophysiological mechanisms involved during kidney allograft rejection, could improve the molecular classifiers for its detection and prediction and provide new and unknown targets for drug designing and repurposing.

In the present study we investigated the discovery capability of Next-Generation Sequencing (NGS) to unravel both coding and non-coding transcriptome differentially expressed during rejection. For that purpose, we built a real-world, multicentric and extensively phenotyped cohort of 540 patients (770 biopsies) from two clinical studies: EU-TRAIN (NCT03652402) and KTD-Innov (NCT03582436). We performed an Illumina sequencing, analyzed the samples with differential gene expression analysis, identified known genes according to published gene panels (microarray or the Banff Human Organ Transplant) to identify new transcripts and implemented pathway enrichment analysis on the different subgroups.

## Material and Methods

### Study Population and Biopsy Cohort

EU-TRAIN (NCT03652402) and KTD-Innov (NCT03582436) studies are large, prospective multicenter cohorts that follow adult kidney transplant recipients for 1 year after transplantation. They involve collaboration between transplant centers, analytical platforms, and industrial partners across France and Europe.

The studies focus on adult patients (18 years or older) receiving a living or deceased kidney transplant. Participants must be willing to comply with study procedures and signed an informed consent. Patients with a history of multi-organ transplants, language barriers hindering participation, or vulnerability (minors, pregnant women, etc.) were excluded.

Both EU-TRAIN and KTD-Innov involve baseline visits at the time of transplant, followed by checkups at 3- and 12-months post-transplantation. Additional visits may be scheduled if a patient’s kidney function deteriorated or protein levels raised. KTD-Innov recruited participants between July 2018 and December 2019 and focused on seven French transplant centers (Paris-Necker, Paris-Saint-Louis, Nantes, Bordeaux, Toulouse, Lyon, and Montpellier). The EU-TRAIN study, with a slightly broader enrollment window (November 2018–June 2020), encompasses nine centers across Europe (Paris-Saint-Louis, Paris-Necker, Nantes, Barcelona-Bellvitge, Barcelona-Vall d’Hebron, Berlin-Charité Mitte, Berlin-Charité Virchow, Geneva, Paris-Kremlin-Bicêtre). 770 renal biopsies were collected from 540 patients from the two prospective studies as well as two retrospective cohorts from Necker and St Louis hospitals (Paris, France) between 2006 and 2021. This study was approved by local institutional review boards and written informed consent was obtained from all patients.

### Kidney Allograft Phenotypes

Lesions from biopsies were graded by local renal specialist from 0 to 3 according to the 2019 international Banff classification [13], and comprised: glomerulitis (g), peritubular capillary inflammation (ptc), interstitial inflammation (i), tubulitis (t), total inflammation (ti), endarteritis (v), transplant glomerulopathy (cg), interstitial fibrosis (ci), tubular atrophy (ct), vascular fibrous intimal thickening (cv), arteriolar hyalinosis (ah). C4d staining was performed by immunohistochemistry on paraffin sections using the human C4d polyclonal antibody. C4d staining was graded from 0 to 3 by the percentage of peritubular capillaries with linear staining. Earlier biopsies were reclassified to take into account the evolution of the classification.

### Detection and Characterization of Circulating Donor-specific anti-HLA Antibodies

The presence of circulating donor-specific anti-HLA-A, -B, -Cw, -DR, -DQ and -DP antibodies was analyzed using single-antigen bead assays (One Lambda, Inc., Canoga Park, CA, United States) on a Luminex platform on serum samples collected at the time of transplantation and at the time of biopsy. For each patient, we recorded the number, class, specificities and mean fluorescence intensity (MFI) of all donor-specific HLA antibodies. Positiveness of a DSA was defined by a threshold of 500 for the mean fluorescence intensity. The maximum MFI for the immunodominant DSA (Anti-HLA iDSA MFI) was defined as the highest ranked donor-specific bead. HLA typing of donors and recipients was performed using DNA typing.

### Experimental Procedures

After collection, all biopsies were stored in the RNAlater^®^ solution at −20°C. They were then centralized at the Paris Cardiovascular Research Center (PARCC) in order to be processed by the Paris Transplant Group Precision Pathology Platform for total RNA extraction using the Promega^®^ Maxwell^®^ RSC miRNA Tissue Kit [14]. All samples were selected according to a minimal concentration of RNA of 20 ng/μL and an RNA integrity number superior or equal to 7. Purified RNAs were, then, stored in a −80°C fridge while waiting to be sent and sequenced by the GENOM’IC platform at Cochin hospital where the library was prepared according to the Illumina^®^ Stranded mRNA Prep Ligation protocol [15] with a capture of the polyadenylated RNAs using oligo (dT) magnetic beads. Finally, an Illumina sequencing has been performed in order to obtain 2 × 30 millions paired-end reads on average.

### RNA-Seq Data Processing and Quality Controls

After the sequencing, we used FastQC (v0.11.9) [16] to assess the pre-alignment quality controls. We performed the alignment with the STAR algorithm (v2.7.4a) [17] and the Hg38.p13 reference genome. We finally verified its quality with STAR, Picard tools (v 2.22.9) [18] and RSeQC (v3.0.1) [19] metrics. Raw counts have been generated using the featureCounts program with the GC_000001405.39_GRCH38.p13 GTF annotation file and the BAM files resulting from the alignment. Quality controls results can be found in the Supplementary Table S1.

### Differential Gene Expression Analysis

The identification of differentially expressed genes (DEGs) was performed using the DESeq2 method (v1.30.1) [20]. Gene expression count matrix has been pre-filtered by removing lowly-expressed genes using a threshold of at least 1 Fragment Per Kilobase Million (FPKM) in 20% of the total samples for each gene. The number of filtered genes reduced from 44,613 to 15,563. Fold changes (FC) and Wald statistics were computed for each comparison of interest with a correction for multiple hypothesis testing (Benjamini-Hochberg) and genes were ranked according to increasing adjusted *p*-values.

Two differential gene expression analysis were conducted including antibody-mediated rejection (AMR) and T-cell-mediated rejection (TCMR). Each diagnosis was tested against all histopathological diagnoses available in the cohort to obtain a molecular signature specific for the diagnosis of interest. This control group included all biopsies diagnosed with either TCMR or AMR (according to the design), isolated interstitial fibrosis and tubular atrophy, acute tubular necrosis, polyomavirus-associated nephropathy, thrombotic microangiopathy, recurrent or *de novo* glomerulonephritis, calcineurin-inhibitor toxicity or biopsies with no evidence of specific lesions (i.e., normal biopsies). Missing information, borderline (N = 69), mixed (N = 20) and suspicious rejection (N = 12) samples have been excluded from both designs. No threshold on the log_2_ fold change was applied and all significant (adjusted *p*-value <0.05) differentially-expressed genes were considered during the analysis.

The complete description of differentially expressed gene symbols, mean expressions, log_2_ fold changes, standard errors and Wald statistics as well as descriptions of the genes previously described in gene panels (B-HOT or microarrays) are shown in the Supplementary Material.

### Pathways Enrichment Analysis

Pathways analysis was performed using both Reactome and the Kyoto Encyclopaedia of Genes and Genomes (KEGG) repositories with ReactomePA (v1.34.0) [21] and clusterProfiler (v3.18.1) [22], respectively, by either choosing as an input the entire list of upregulated genes (Reactome and KEGG) or a subset consisting of the upregulated transcripts included in the B-HOT or the microarray gene panel (Reactome only). Raw *p*-values were corrected for multiple hypothesis testing with the Benjamini-Hochberg FDR controlling technique and two cut-offs were applied to filter non-significant results: threshold of 0.05 on the adjusted *p*-value and 0.2 on the q-value. Q-values correspond to the proportion of false positive results in a set of signaling pathways that are at least as significant (adjusted *p*-value) as the signaling pathway under consideration. While the adjusted *p*-value gives the expected false positive rate, the q-value gives the expected positive false discovery rate. Pathway names, annotations and statistics are reported in the Supplementary Material.

### Statistical Analysis

Continuous variables were described by using means and standard deviations or medians and interquartile ranges. All analyses were performed using R (version 4.0.5, R Foundation for Statistical Computing). Values of *p* < 0.05 were considered significant, and all tests were 2-tailed.

## Results

### Characteristics of the Population

The study cohort comprised a total of 770 kidney allograft biopsies from 540 patients collected between 2006 and 2021 from 11 international European centers (See Supplementary Material). Baseline characteristics including recipient and donor characteristics are summarized in Table 1. The population was mainly composed of men (n = 336, 63.2%) with a mean age of 51.1 ± 15.9 years at the time of transplantation, a history of glomerulonephritis as end stage renal disease (n = 125, 23.2%) and no history of a prior kidney transplant (n = 435, 80.9%). The majority of the transplantations were performed from deceased donors (n = 439, 81.5%) with 185 (42.1%) exhibiting expanded criteria. In total, 141 (27.9%) patients had pre-existing anti-HLA DSA.

**TABLE 1:** Baseline patient characteristics.

	NGS cohort (n = 540)	N
Recipient characteristics		
Age (years), Mean (SD)	51.1 (15.9)	540
Gender male, No. (%)	336 (63.2)	532
End stage renal disease causes		539
ADPKD, No. (%)	82 (15.2)	
Diabetes, No. (%)	48 (8.9)	
Glomerulonephritis, No. (%)	125 (23.2)	
Tubulo-interstitial, No. (%)	58 (10.8)	
Vascular, No. (%)	60 (11.1)	
Other, No. (%)	79 (14.7)	
Unknown, No. (%)	87 (16.1)	
Donor characteristics		
Age (years), Mean (SD)	54.4 (17.1)	534
Gender male, No. (%)	298 (55.7)	535
Hypertension, No. (%)	144 (28.6)	504
Diabetes, No. (%)	40 (7.8)	513
Creatinine (µmol/L), Mean (SD)	83.8 (51.0)	530
Donor type		
Living donor, No. (%)	100 (18.5)	539
Deceased donor, No. (%)	439 (81.5)	539
Expanded criteria donor, No. (%)	185 (42.1)	439
Transplant baseline characteristics		
Prior kidney transplant, No. (%)	103 (19.1)	538
Cold ischaemia time (hours), Mean (SD)	13.9 (8.4)	534
Delayed graft function, No. (%)	77 (14.5)	531
HLA-A/B/DR/DQ mismatch, Median (IQR)	5 (4–6)	465
Presence of D0 DSA, No. (%)	141 (27.9)	505

Delayed graft function was defined as the use of dialysis in the first postoperative week. Abbreviations: ADPKD: autosomal dominant polycystic kidney disease; DSA: donor specific antibody; HLA: human leucocyte antigen.

The median time from transplantation to the biopsy was 3.9 months (IQR: 3.0–12.1) (Supplementary Figure S1) with 460 (60%) protocol biopsies and 370 (40%) for cause biopsies. The mean number of biopsy per patient was 1.4 (median = 1), with a maximum of 5 biopsies per patient. The mean eGFR at the time of the biopsy was 42.8 ± 19.1 mL/min/11.73 m^2^ with a mean proteinuria of 0.53 ± 1.58 g/g. One-third of the patients (n = 226, 31.3%) had positive anti-HLA DSA with the immunodominant DSA belonging mainly to the class II (n = 148, 67.6%) (Table 2).

**TABLE 2:** Histological, immunological and functional characteristics at the time of biopsy.

	Included samples (n = 770)	N
Histological characteristics		
Time since transplantation (months), Median (IQR)	3.90 [2.97; 12.1]	770
Banff scores		
g score > 0, No. (%)	109 (14.8)	737
ptc score > 0, No. (%)	133 (18.2)	730
i score > 0, No. (%)	141 (19.1)	738
t score > 0, No. (%)	203 (27.5)	739
v score > 0, No. (%)	31 (3.8)	697
cg score > 0, No. (%)	47 (6.4)	734
cv score > 0, No. (%)	445 (66.4)	670
ci score > 0, No. (%)	420 (57.1)	736
ct score > 0, No. (%)	435 (59.2)	734
ti score > 0, No. (%)	206 (29.6)	696
i-IFTA score > 0, No. (%)	117 (18.7)	626
t-IFTA score > 0, No. (%)	13 (4.2)	312
ah score > 0, No. (%)	416 (57.8)	721
aah score > 0, No. (%)	40 (22.9)	175
mm score > 0, No. (%)	59 (8.3)	709
C4d score > 0, No. (%)	108 (15.2)	710
Diagnosis according to pathologist		
Normal, No. (%)	152 (20.6)	738
Borderline, No. (%)	69 (9.4)	737
T-cell mediated rejection, No. (%)	72 (9.9)	724
Antibody-mediated rejection, No. (%)	88 (12.0)	736
IFTA positive, No. (%)	365 (49.6)	736
Recurrent nephropathy, No. (%)	15 (2.0)	754
*De novo* glomerulonephritis, No. (%)	18 (2.4)	752
Acute tubular necrosis, No. (%)	68 (9.1)	749
Polyomavirus nephropathy, No. (%)	30 (4.0)	748
CNI toxicity, No. (%)	65 (8.7)	748
Thrombotic microangiopathy, No. (%)	34 (4.6)	748
Immunological characteristics		
Anti-HLA DSA, No. (%)	226 (31.3)	722
Anti-HLA DSA class		221
I, No. (%)	43 (19.5)	
II, No. (%)	114 (56.1)	
I and II, No. (%)	64 (29.0)	
Anti-HLA iDSA MFI, Mean (SD)	3,229 (4,060)	219
Functional characteristics		
Proteinuria (g/g), Median (IQR)	0.20 [0.10; 0.41]	736
eGFR (MDRD), Mean (SD)	42.8 (19.1)	727

eGFR, was calculated according to the MDRD, formula without the 1.21 ethnicity and 0.94 standardized creatinine factors. Abbreviations: (i) DSA: (immunodominant) donor specific antibody; eGFR: estimated glomerular filtration rate; HLA: human leucocyte antigen; IFTA: interstitial fibrosis and tubular atrophy; MFI: mean fluorescence intensity.

### Histological Phenotypes

Kidney allograft biopsies were either classified as normal (n = 152, 20.6%) or had histological evidence for one or multiple of the following diagnoses: T-cell mediated rejection (n = 72, 9.9%), antibody-mediated rejection (n = 88, 12.0%), mixed rejection 14 (10.6%), borderline rejection (n = 69, 9.4%), interstitial fibrosis and tubular atrophy (n = 365, 49.6%), recurrence of the initial nephropathy (n = 15, 2.0%), *de novo* or recurrent glomerulonephritis (n = 18, 2.4%), acute tubular necrosis (n = 68, 9.1%), polyomavirus-associated nephropathy (n = 30, 4.0%), calcineurin inhibitors-related toxicity (n = 65, 8.7%), and thrombotic microangiopathy (n = 34, 4.6%) (Table 2).

### Molecular Landscape of Antibody-Mediated Rejection

**FIGURE 1:**
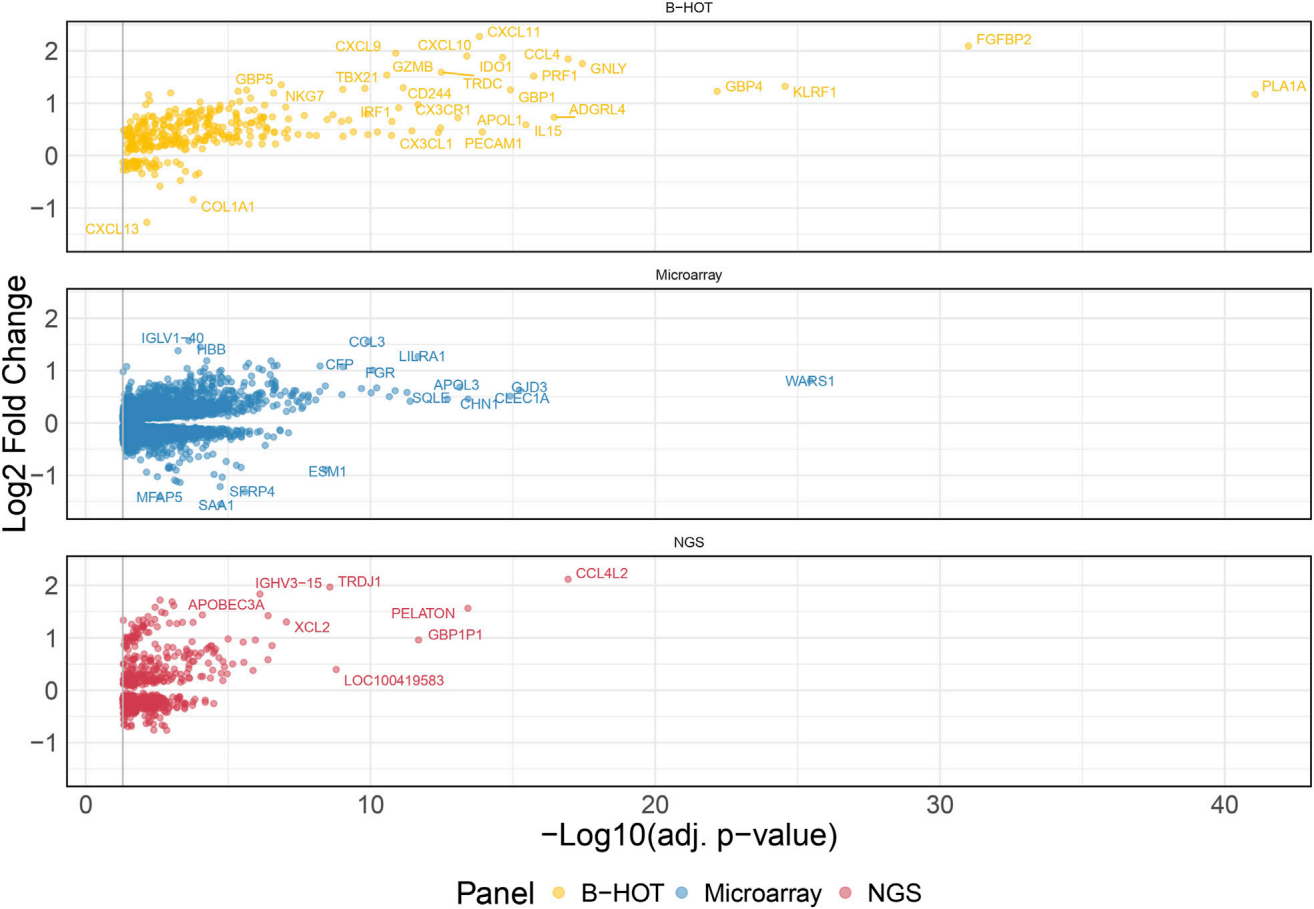
Antibody-mediated rejection molecular signature. Volcano plot of the significant differentially expressed genes associated with antibody-mediated rejection. Dots are related to each gene. The significant transcripts are displayed according to a 0.05 threshold on the adjusted *p*-value (vertical grey line). NGS-specific transcripts are highlighted in red, B-HOT-related in yellow and microarray-related in blue. *X*-axis represents the -log_10_ of the adjusted *p*-value (the higher, the smaller is the *p*-value) and the *y*-axis represents the log_2_ fold change. Differences in gene expression between the AMR and non-AMR group are marked with positive (negative) values correspond to up- (down-)regulated transcripts in the AMR group. In total, 6,141 genes were differentially expressed showing the following distribution: 358 included in the B-HOT gene panel, 5,180 included in the microarray gene panel and 603 NGS-specific. Abbreviations: AMR: antibody-mediated rejection; B-HOT: Banff Human Organ Transplant; NGS: next-generation sequencing.

60 AMR were compared to 576 non-AMR samples, resulting in 6,141 differentially expressed genes (DEGs). 358 (5.8%) were included in the Banff Human Organ Transplant (B-HOT) gene panel, 5,180 (84.4%) were included in the microarray gene panel, and 603 (9.8%) were new and defined as NGS-specific transcripts (Figure 1 and Supplementary Material). Genes included in the microarrays were highly represented throughout the entire molecular signature (from 0.0% to 84.4% among the increasing top ranked genes and stabilizing at top 2,500 genes), while the B-HOT-related genes were mainly ranked in the top genes (from 100.0% to 5.8%%, reaching 47.0% among the top 100 genes, 25.4% among the top 500 genes and 17.0% among the top 1,000 genes), and the new NGS-specific genes were constantly comprised across the signature between 5.2% and 12.5% of the total (Supplementary Figure S2). Among the top 30 ranked genes, 20 genes (66.7%) were included in the B-HOT gene panel including *PLA1A*, *GBP1/4*, *GNLY*, *CCL4*, *IL15*, *IDO1*, *CXCL10/11*, 7 (23.3%) genes were included in the microarrays panel (*WARS1*, *GJD3*, *CLEC1A*, *CHN1*, *APOL3*, *SQLE*, *LILRA1*), and 3 genes (10%) were specific to the NGS gene panel with *CCL4L2*, *PELATON* (a long non-coding RNA) and *GBP1P1* (Supplementary Table S2).

**FIGURE 2:**
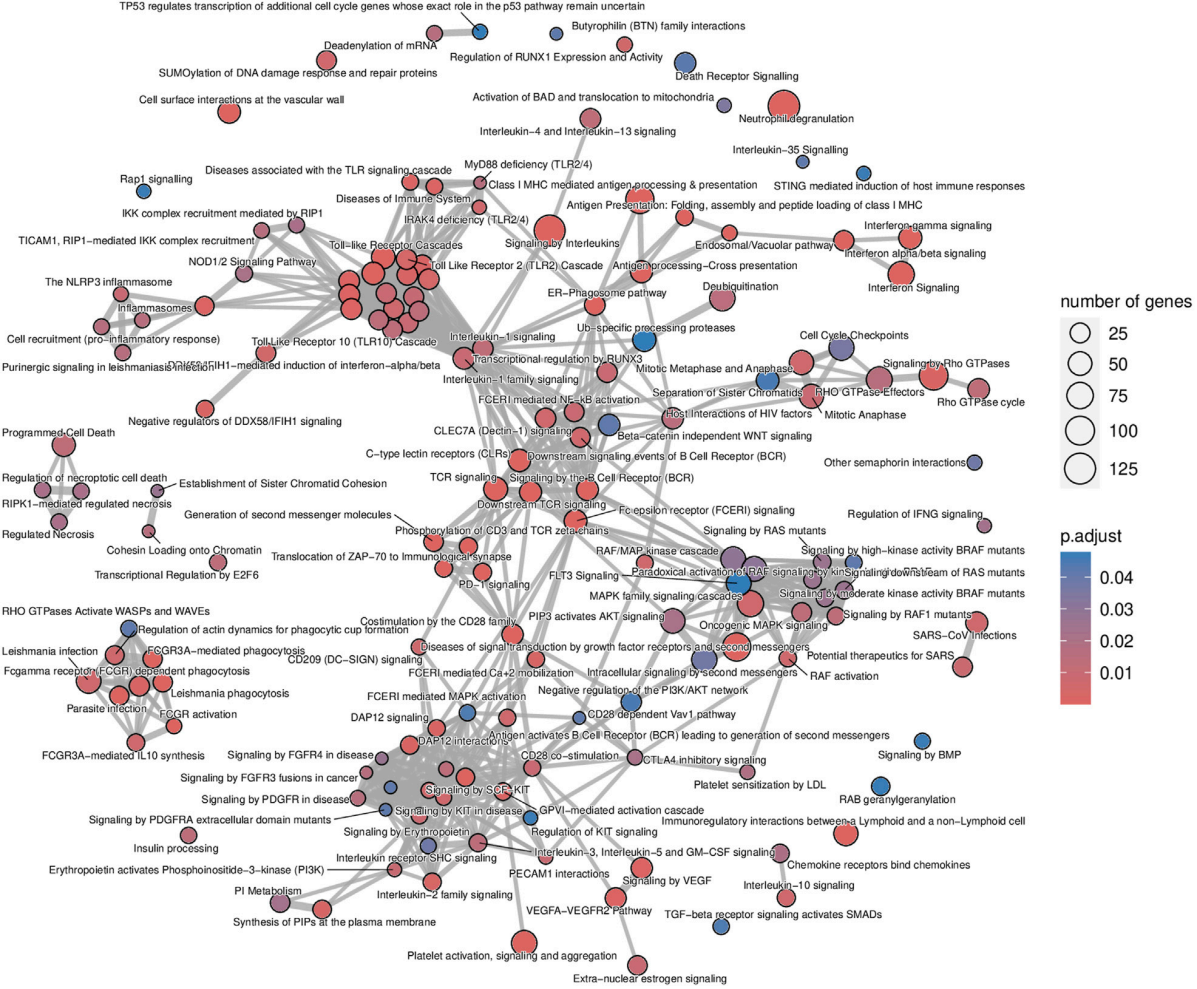
Antibody-mediated rejection map of enriched pathways. Enrichment map of pathways involved in the antibody-mediated rejection, developed on the entire list of upregulated genes. The interaction map contextualizes the pathophysiological categories inter-relations. Vertices represent pathways, dots color intensity refers to the significance of the category and sized of the dots is in accordance to the number of genes in the signature. Edges symbolize the overlap between two pathways, powered by the shared transcripts. The closer two vertices are and the thicker is the edge connecting them, the wider is the overlap between the two pathways.

The list of upregulated and differentially expressed genes was composed of 2,876 genes from which 2,299 (79.9%) were included in the microarrays, 313 (10.9%) were included in the B-HOT panel, and 264 (9.2%) were NGS-specific. Pathway analysis was performed using the Reactome repository. Top ranked (adj.*p*-value<0.05) known pathological categories were related to immune response: interferon signaling (q-value = 1.78e^−11^), neutrophil degranulation (q-value = 4.57e^−11^), signaling by interleukins (q-value = 4.11e^−10^), Toll-like receptors cascades (q-value = 6.80e^−06^), class I MHC mediated antigen processing and presentation (q-value = 1.37e^−05^), Fc Gamma/Fc Epsilon receptors (q-value = 2.18e^−04^ and q-value = 2.15e^−05^, respectively), signaling by the BCR (q-value = 5.40e^−05^), cell surface interactions at the vascular wall (q-value = 2.11e^−04^) and PECAM1 interactions (q-value = 8.14e^−03^). In addition, the TCR signaling (q-value = 1.78e^−11^), the PD-1 signaling (q-value = 3.50e^−08^), the CD28 co-stimulation (q-value = 7.43e^−03^) and the CTLA4 inhibitory signaling (q-value = 1.78e^−02^) pathways were found significantly enriched in the AMR signature (Figure 2; Supplementary Figure S3 and Supplementary Material). Enrichment analysis derived from the KEGG database demonstrated additional significant pathways including the NK cell mediated cytotoxicity (q-value = 2.44e^−10^), Th17 cell differentiation (q-value = 2.98e^−10^) and Th1 and Th2 cell differentiation (q-value = 4.97e^−07^), and provided access to the entire set of cytokine and receptors (CCL4, CCL11, CXCL5/6/9/10/11, XCL2, IL15/16/27/34/35, TNF, TGFβ) and cell adhesion and endothelium-related molecules (CD58, MHCI/II, CD40, ITGA, CD2, CD4, PD-L1, CDH5, PECAM1) involved during antibody-mediated rejection (Supplementary Figures S4 and S5 and Supplementary Material).

The analysis of enriched pathways restrained to the different panels highlighted specific functions. The B-HOT panel captured all the above-mentioned significant functions with a total of 191 entries in Reactome (Supplementary Figure S6 and Supplementary Material) while the microarray panel was specifically enriched in SUMOylation processes, RHO/RAC GTPase cycle, cell cycle progression and FCGRIIIA-mediated phagocytosis with only 44 entries (Supplementary Figure S7 and Supplementary Material). Finally, despite its 264 upregulated DEGs, the NGS-specific genes were only enriched in 4 non-specific metabolic functions (Supplementary Figure S8).

### Molecular Landscape for T-cell Mediated Rejection

**FIGURE 3:**
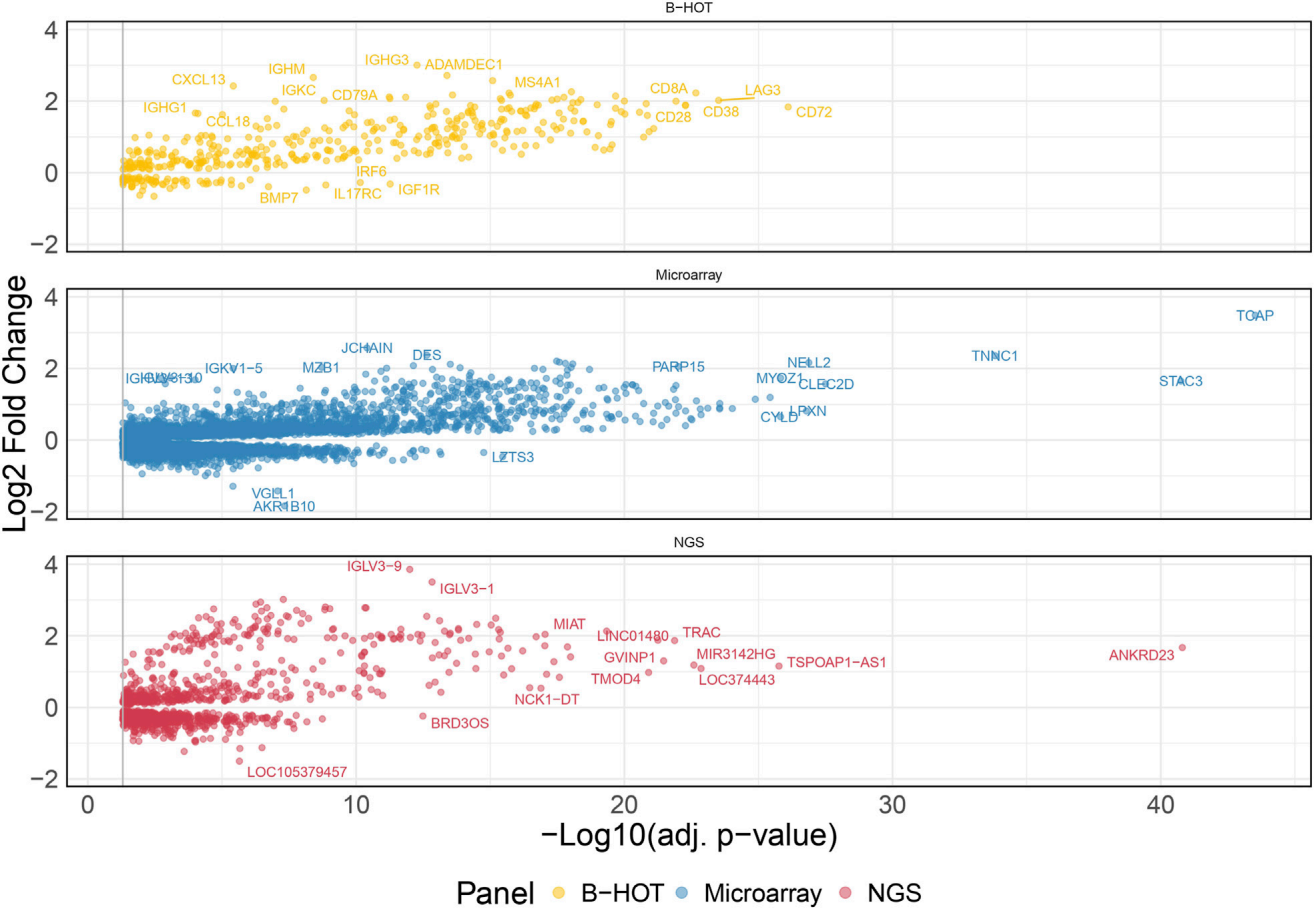
T-cell mediated rejection molecular signature. Volcano plot of the significant differentially expressed genes associated with T-cell mediated rejection. Dots are related to each gene. The significant transcripts are displayed according to a 0.05 threshold on the adjusted *p*-value (vertical grey line). NGS-specific transcripts are highlighted in red, B-HOT-related in yellow and microarray-related in blue. *X*-axis represents the -log_10_ of the adjusted *p*-value (the higher, the smaller is the *p*-value) and the *y*-axis represents the log_2_ fold change. Differences in gene expression between the TCMR and non-TCMR group are marked with positive (negative) values correspond to up- (down-)regulated transcripts in the TCMR group. In total, 8,478 genes were differentially expressed showing the following distribution: 439 included in the B-HOT, 6,853 included in the microarray and 1,186 NGS-specific. Abbreviations: B-HOT: Banff Human Organ Transplant; NGS: next-generation sequencing; TCMR: T-cell mediated rejection.

48 TCMR were compared to 589 non-TCMR samples and the molecular signature was defined by 8,478 genes. 439 (5.2%) were included in the B-HOT panel, 6,853 (80.8%) were included in the microarrays and 1,186 (14.0%) were NGS-specific (Figure 3). After ranking genes by their adjusted *p*-value, the proportions of transcripts included with each gene panel were mostly in favor of the microarray panel (from 100.0% to 80.8% with a local minimum of 60.8% among the top 265 genes). The proportion of genes included in the B-HOT first increased to reach a maximum of 30.5% among the top 118 genes, before decreasing to reach a minimum of 5.1%. Except among the top 5 genes, the newly discovered NGS genes were relatively stable (between 7.6% and 18.1%) (Supplementary Figure S9). Among the top 30 ranked genes, 22 (73.4%) were comprised in the microarray gene panel, 4 (13.3%) were comprised in the B-HOT panel (*CD72*, *LAG3*, *CD8A*, *CD28*) and 4 (13.3%) were NGS-specific (*ANKRD23*, *TSPOAP1-AS1*, *LOC374443*, *MIR3142HG*) (Supplementary Table S3).

**FIGURE 4:**
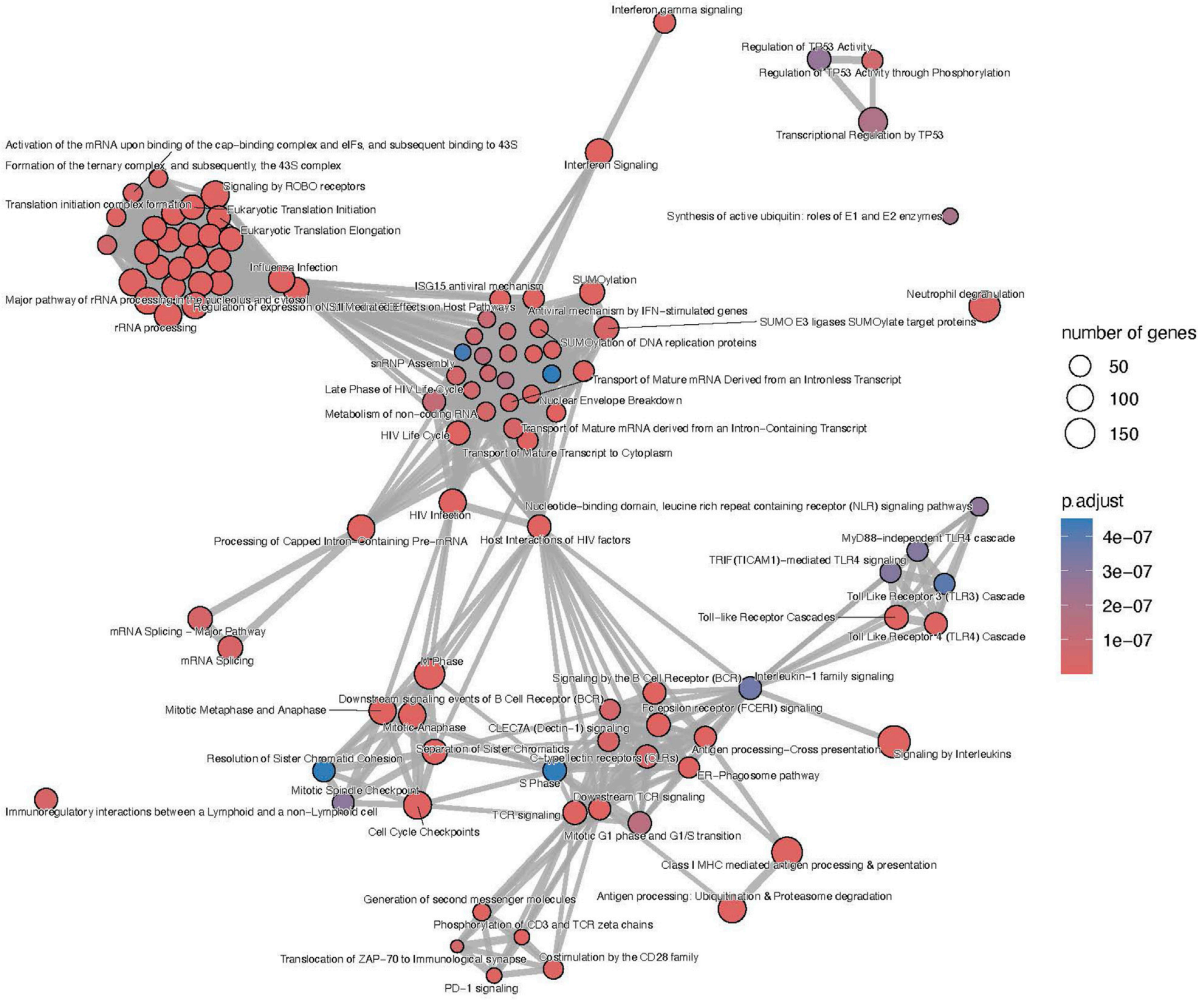
T-cell mediated rejection map of enriched pathways. Enrichment map of pathways involved in the antibody-mediated rejection, developed on the entire list of upregulated genes. The interaction map contextualizes the pathophysiological categories inter-relations. Vertices represent pathways, dots color intensity refers to the significance of the category and sized of the dots is in accordance to the number of genes in the signature. Edges symbolize the overlap between two pathways, powered by the shared transcripts. The closer two vertices are and the thicker is the edge connecting them, the wider is the overlap between the two pathways. Only the top 100 pathways are displayed to improve readability.

The list of upregulated differentially expressed genes was composed of 4,482 genes from which 3,612 (80.6%) were included in the microarrays, 367 (8.2%) were included in the B-HOT panel and 503 (11.2%) were NGS-specific. Using the entire list of upregulated genes, significantly immunological Reactome enriched pathways comprised pathways related to: interferon signaling (q-value = 1.76e^−22^), signaling by ROBO receptors (q-value = 2.66e^−22^), TCR signaling (q-value = 2.40e^−17^), class I MHC mediated antigen processing and presentation (q-value = 1.88e^−16^), signaling by interleukins (q-value = 2.42e^−16^), signaling by the BCR (q-value = 8.04e^−13^), Fc Epsilon receptor signaling (q-value = 1.79e^−11^) and Fc Gamma receptor dependent phagocytosis (q-value = 1.09e^−04^), TLR cascades (q-value = 9.18e^−10^), co-stimulation by the CD28 family (q-value = 3.88e^−09^), PD-1 signaling (q-value = 4.14e^−09^) and neutrophil degranulation (q-value = 6.69e^−09^). Out of the 466 Reactome entries, emphasis was also given on nonsense mediated decay and maturation of mRNA functions, SUMOylation processes, metabolism of non-coding RNA and cell cycle progression (Figure 4 and Supplementary Material). The KEGG repository significantly presented enrichment of the Th17 cell differentiation (q-value = 4.72e^−14^), Th1 and Th2 cell differentiation (q-value = 8.13e^−12^), and NK cell mediated cytotoxicity (q-value = 1.10e^−08^). A wider range of activation/inhibition of cell adhesion molecules was also presented with lower/higher fold changes (min = −1.8, max = 3.9) compared to the AMR signature. The TCMR signature included the addition of CD22, PDCD1 and SELL and the inhibition of a multitude of molecules at the surface of the endothelial cells (Supplementary Figure S10 and Supplementary Material).

Focusing on the different gene panels, the genes included in the B-HOT panel captured all the immunological functions described previously (Supplementary Figure S11 and Supplementary Material) while the microarray genes specifically captured the nonsense mediated decay, SUMOylation, translation and mRNA maturation processes and the cell cycle progression (Supplementary Figure S12 and Supplementary Material). Finally, with 503 upregulated genes, no enriched pathways were annotated for the new NGS-specific genes.

## Discussion

In this study we aimed at defining and describing the molecular profiles and biological functions associated with antibody-mediated and T-cell mediated rejection, combining a deeply phenotyped cohort of kidney allograft biopsies and next-generation sequencing analyses. For this purpose, we used the histological labels and the gene expressions as inputs for a differential expression analysis and ranked the significant genes according to the adjusted *p*-values. We, then, queried publicly available biological databases to understand the pathophysiological mechanisms derived from the upregulated DEGs. In this study, an emphasis was made to discriminate genes from known gene panels (B-HOT and microarray) already validated and used in clinical practice [23–26] and new genes discovered using the NGS technology.

In the present study, active antibody-mediated rejection was found in 9.4% of the analyzed samples. This incidence aligns well with the most recently reported incidence of AMR ranging from 3% to 12% in a recent systematic review including 28 studies [27]. Its molecular signature included features of macrophages activation (*CD40*, *CD58, IDO1*), NK cells activation (*GNLY*, *FGFBP2*, *CD16a*), cytotoxic T cells activation (*CD8*), helper T cells activation (*CD4*), endothelial cells activation (*ICAM1*, *PECAM1*, *VCAM1*, *CDH5*), and B cells activation (*CD22*, *CD40*, *CD86*), which showed great consistency with the microarray studies [28, 29]. From both innate and adaptive immune systems, the enrichment analysis confirmed the ability of the B-HOT gene panel to capture both components occurring during rejection but presented a lack of metabolic functions, such as SUMOylation processes and cell cycle progression and checkpoint that are specifically present in the microarrays. Regarding the NGS-specific gene panel, 603 new genes (comprising 264 upregulation) were found associated with AMR but no annotation was available in the public repositories. They were mainly composed of long non-coding RNAs that are poorly described in the literature. *PELATON*, for instance, was part of the new NGS-specific top ranked genes and was found to be a regulator specifically located in macrophages and monocytes nucleus, for which the downregulation is associated to decreased phagocytosis functions [30]. In this study, we found that *PELATON* was upregulated during AMR (log_2_FC = 1.56), in line with a probable increased phagocytosis function occurring in the microcirculation inflammation and, consequently, potentially leading to increased differentiation into antigen-presenting cells, T-cell recruitment and activation and, ultimately, B-cell proliferation and transformation into plasma cells.

Compared to the AMR signature, the TCMR signal presented a similar profile compared to the published studies in terms of genes (*CD72*, *LAG3*, *CD8A*, *CD28*, *ANKRD* family) and activated cell types and functions [31]. However, a key difference existed in the repertoire of inhibited cell adhesion molecules, showing strong inhibition of the endothelial and epithelial cells receptors, emphasizing the cell infiltration observed at the histological level. Regarding the different gene panels, the microarray was specific of mRNA maturation processes and nonsense mediated decay, which could be due to a lack of annotation of the different repositories. In our study, the B-HOT panel was enriched by the main immunological functions but did not include the top adjusted *p*-value ranked genes, potentially limiting its ability to accurately diagnose TCMR. The addition of new genes, for example, from the microarray or discovered with the NGS technology, could potentially help the molecular classifiers. Finally, for the NGS-specific markers, they were composed of lncRNA which lacked annotation in the current repositories. Few of them are described in the literature such as MIR3142HG which was shown to be a positive regulator of IL-8 and CCL2 [32].

The main advantage of the present study is that the cohort’s diverse phenotypes encompass most of the clinical scenarios encountered in routine practice. It also gathered samples and patients representing a real-life setting in terms of population demographics, rejection prevalence and immunosuppression therapies. Lastly, this is, to our knowledge, the first RNA-seq experiment applied in such cohort characteristics (size, heterogeneity, description) to study the molecular signature of rejection in kidney allograft biopsies. A literature review on PubMed comprising the key words “NGS,” “transplantation,” “kidney” and “rejection” resulted in 46 articles published over the last 5 years: 11 (23.4%) were related to cell-free DNA, 9 (19.6%) were related to infections (comprising also BK virus), 4 (8.7%) were focusing on cell subpopulations, 5 (10.9%) were related to response to treatment and 5 (10.9%) were related to HLA matching. Five references mentioned either the use of NGS or the B-HOT gene panels but showed limitations in the number of patients/biopsies, number of genes under study, in their design (sick vs. well, single centre), or in the representativity of the different diagnoses [33–37].

Regarding the study limitations, one of the main issues is the sampling bias regarding the technique requiring an extra core. The sequenced core might be different from the one analyzed by the pathologist both in terms of quality (i.e., number of glomeruli and arteries) and severity of the disease, which is not the case for the Nanostring technology and the B-HOT gene panel where an extra core is not needed. Second, while NGS might help to discover new genes and physio-pathological pathways, its use in clinical practice is limited in terms of access to the technology and its cost. In our study, most of the genes associated with AMR and TCMR were included in the microarray and the B-HOT-gene panels, validating the relevance and the accuracy of the genes included. Finally, from a clinical aspect, our cohort was mainly treated with corticosteroids, mycophenolic acid and tacrolimus, which might have an impact on the observed molecular expressions. The presented results should be validated on patients treated with different types of immunosuppressive therapies including mTOR inhibitors or Belatacept.

## Conclusion

We discovered 9.8% and 14.0% novel transcripts associated with antibody-mediated rejection and T-cell mediated rejection, respectively. The main immunological functions were positively captured by both the microarray and B-HOT gene panels. Those new NGS specific transcripts might represent a novel source of targets for drug designing and repurposing.

## Data Availability

The complete list of activated pathways and differentially expressed genes are available in the synapse public Synapse database (https://www.synapse.org/Synapse:syn60959798/files/).

## Supplementary Material

Supplementary Data Sheet S1
AMR signature.

Supplementary Data Sheet S2
TCMR signature.

Supplementary Data Sheet S3
AMR (B-HOT panel) Reactome pathways.

Supplementary Data Sheet S4
AMR (microarray panel) Reactome pathways.

Supplementary Data Sheet S5
AMR Reactome pathways.

Supplementary Data Sheet S6
TCMR (B-HOT panel) Reactome pathways.

Supplementary Data Sheet S7
TCMR (microarray panel) Reactome pathways.

Supplementary Data Sheet S8
TCMR Reactome Pathways.

Supplementary Data Sheet S9
AMR KEGG pathways.

Supplementary Data Sheet S10
TCMR KEGG pathways.

Supplementary Data Sheet S11
Supplementary Appendix.

## Abbreviations

AMR, antibody-mediated rejection; B-HOT, Banff Human Organ Transplant; DEGs, differentially expressed genes; DSA, donor-specific antibodies; FC, fold change; FDR, false discovery rate; FPKM, fragments per kilobase of exons per million fragments; NGS, next-generation sequencing; TCMR, T-cell-mediated rejection.
