# Supplementary material for: Antibody Mediated Rejection and T-cell Mediated Rejection Molecular Signatures Using Next-Generation Sequencing in Kidney Transplant Biopsies
Source: Transpl Int. 2024 Jul 10;37:13043. doi: 10.3389/ti.2024.13043 (PMC11267505; doi:10.3389/ti.2024.13043)
Supplement: Supplementary file 8 [file DataSheet11.docx]

**Supplementary appendix**

1. **SUPPLEMENTARY METHODS**
   1. **Cohort description**
2. **SUPPLEMENTARY TABLES**

**Supplementary Table 1: Sequenced samples quality controls statistics**

**Supplementary Table 2: Top *3*0 adjusted p-value ranked differentially expressed genes associated with antibody-mediated rejection**

**Supplementary Table 3:** **Top 20 adjusted p-value ranked differentially expressed genes associated with T-cell mediated rejection**

1. **SUPPLEMENTARY FIGURES**

**Supplementary Figure 1: Distribution of the time from transplantation**

**Supplementary Figure 2: Gene panels distribution across the antibody-mediated rejection signature**

**Supplementary Figure 3: Top 30 overall enriched pathways in antibody-mediated rejection**

**Supplementary Figure 4: Set of cytokines and cytokine receptors engaged in the antibody-mediation rejection signature**

**Supplementary Figure 5: Set of cell adhesion molecules engaged in the antibody-mediation rejection signature**

**Supplementary Figure 6: Antibody-mediated rejection map of enriched pathways (BHOT-specific up-regulated gene list)**

**Supplementary Figure 7: Antibody-mediated rejection map of enriched pathways (Microarray-specific up-regulated gene list)**

**Supplementary Figure 8: Antibody-mediated rejection map of enriched pathways (NGS-specific up-regulated gene list)**

**Supplementary Figure 9: Gene panels distribution across the T-cell mediated rejection signature**

**Supplementary Figure 10: Set of cell adhesion molecules engaged in the T-cell mediation rejection signature**

**Supplementary Figure 11: T-cell mediated rejection map of enriched pathways (BHOT-specific up-regulated gene list)**

**Supplementary Figure 12: T-cell mediated rejection map of enriched pathways (microarray-specific up-regulated gene list)**

1. **SUPPLEMENTARY METHODS**
   1. **Cohort description**

A total of 770 biopsy samples (540 patients) were collected between 2006 and 2021 and were included in the study from 11 European centres.

| **Centre** | **N** |
| --- | --- |
| Necker hospital, Paris (France) | 245 |
| Saint Louis hospital, Paris (France) | 200 |
| Toulouse (France) | 83 |
| Bordeaux (France) | 78 |
| Nantes (France) | 59 |
| Montpellier (France) | 45 |
| Lyon (France) | 39 |
| Bellvitge hospital, Barcelona (Spain) | 12 |
| Charité-Virchow hospital, Berlin (Germany) | 4 |
| Kremlin-Bicêtre, Paris (France) | 3 |
| Geneva (Switzerland) | 2 |

**SUPPLEMENTARY TABLES**

**Supplementary Table 1: Sequenced samples quality controls statistics**

|  | **Included samples**  **(n770)** | **N** |
| --- | --- | --- |
| **Pre-alignment quality controls** |  |  |
| **Mean GC**, Mean (SD) | 49.0 (1.15) | 770 |
| **Per base mean quality**, Mean (SD) | 33.6 (0.37) | 770 |
| **First 10 base mean quality**, Mean (SD) | 32.7 (0.24) | 770 |
| **Last 15 base mean quality**, Mean (SD) | 32.8 (0.55) | 770 |
| **Pct of reads whose Phred mean is under 30**, Mean (SD) | 0.16 (0.04) | 770 |
| **Max N-content per base**, Mean (SD) | 0.13 (0.12) | 770 |
| **Pct of reads with expected length**, Mean (SD) | 100 (0.00) | 770 |
| **Overrepresented sequences (max%)**, Mean (SD) | 0.78 (0.79) | 770 |
| **Post-alignment quality controls** |  |  |
| **Pct of uniquely mapped fragments**, Mean (SD) | 88.7 (3.38) | 770 |
| **Number of uniquely mapped fragments**, Mean (SD) | 30,384,390 (8,021,722) | 770 |
| **Pct of multiple mapped fragments**, Mean (SD) | 5.07 (1.29) | 770 |
| **Number of multiple mapped fragments**, Mean (SD) | 1,736,647 (635,329) | 770 |
| **Junctions by fragment**, Mean (SD) | 0.40 (0.04) | 770 |
| **Pct of coding bases**, Mean (SD) | 40.2 (4.09) | 770 |
| **Pct of intronic bases**, Mean (SD) | 9.59 (1.94) | 770 |
| **Pct of intergenic bases**, Mean (SD) | 25.0 (5.92) | 770 |
| **Pct of ribosomal bases**, Mean (SD) | 20.8 (25.6) | 770 |
| **Pct of UTR bases**, Mean (SD) | 24.4 (2.36) | 770 |
| **Pct of strand-specific bases**, Mean (SD) | 99.3 (0.24) | 770 |
| **Ratio 5'/3'**, Mean (SD) | 1.20 (0.72) | 770 |
| **Pct of known junction**, Mean (SD) | 80.2 (2.81) | 770 |
| **Pct of fragments genes more 1%**, Mean (SD) | 5.03 (2.62) | 770 |
| **Number of genes with FPKM>1**, Mean (SD) | 12,840 (606) | 770 |
| **Median insert size**, Mean (SD) | -7.23 (10.6) | 770 |

**Supplementary Table 2: Top 30 adjusted p-value ranked differentially expressed genes associated with antibody-mediated rejection**

| **Gene symbol** | **Rank** | **Log_2_ FC** | **Adj. p-value** | **Panel** |
| --- | --- | --- | --- | --- |
| ***PLA1A*** | 1 | 1.17 | 8.51e-42 | B-HOT |
| ***FGFBP2*** | 2 | 2.09 | 1.01e-31 | B-HOT |
| ***WARS1*** | 3 | 0.80 | 3.62e-26 | MICROARRAY |
| ***KLRF1*** | 4 | 1.32 | 2.75e-25 | B-HOT |
| ***GBP4*** | 5 | 1.23 | 6.72e-23 | B-HOT |
| ***GNLY*** | 6 | 1.76 | 3.62e-18 | B-HOT |
| ***CCL4*** | 7 | 1.84 | 1.15e-17 | B-HOT |
| ***CCL4L2*** | 8 | 2.12 | 1.15e-17 | NGS |
| ***ADGRL4*** | 9 | 0.73 | 3.56e-17 | B-HOT |
| ***PRF1*** | 10 | 1.52 | 1.85e-16 | B-HOT |
| ***IL15*** | 11 | 0.59 | 3.49e-16 | B-HOT |
| ***GJD3*** | 12 | 0.623 | 5.92e-16 | MICROARRAY |
| ***GBP1*** | 13 | 1.26 | 1.21e-15 | B-HOT |
| ***CLEC1A*** | 14 | 0.51 | 1.25e-15 | MICROARRAY |
| ***IDO1*** | 15 | 1.88 | 2.29e-15 | B-HOT |
| ***PECAM1*** | 16 | 0.45 | 1.19e-14 | B-HOT |
| ***CXCL11*** | 17 | 2.28 | 1.49e-14 | B-HOT |
| ***CHN1*** | 18 | 0.46 | 3.76e-14 | MICROARRAY |
| ***PELATON*** | 19 | 1.56 | 3.79e-14 | NGS |
| ***CXCL10*** | 20 | 1.9 | 4.13e-14 | B-HOT |
| ***APOL3*** | 21 | 0.68 | 7.34e-14 | MICROARRAY |
| ***APOL1*** | 22 | 0.72 | 8.57e-14 | B-HOT |
| ***SQLE*** | 23 | 0.46 | 1.98e-13 | MICROARRAY |
| ***TRDC*** | 24 | 1.59 | 3.26e-13 | B-HOT |
| ***GNG11*** | 25 | 0.53 | 3.46e-13 | B-HOT |
| ***CX3CL1*** | 26 | 0.44 | 4.14e-13 | B-HOT |
| ***GPB1P1*** | 27 | 0.96 | 2.06e-12 | NGS |
| ***CX3CR1*** | 28 | 0.97 | 2.15e-12 | B-HOT |
| ***LILRA1*** | 29 | 1.27 | 2.15e-12 | MICROARRAY |
| ***TM4SF18*** | 30 | 0.47 | 3.51e-12 | B-HOT |

**Supplementary Table 3: Top 30 adjusted p-value ranked differentially expressed genes associated with T-cell mediated rejection**

| **Gene symbol** | **Rank** | **Log_2_ FC** | **Adj. p-value** | **Panel** |
| --- | --- | --- | --- | --- |
| ***TCAP*** | 1 | 3.49 | 3.02e-44 | MICROARRAY |
| ***ANKRD23*** | 2 | 1.67 | 1.57e-41 | NGS |
| ***STAC3*** | 3 | 1.65 | 1.78e-41 | MICROARRAY |
| ***TNNC1*** | 4 | 2.36 | 1.5e-34 | MICROARRAY |
| ***CLEC2D*** | 5 | 1.57 | 3.15e-28 | MICROARRAY |
| ***NELL2*** | 6 | 2.17 | 1.33e-27 | MICROARRAY |
| ***LPXN*** | 7 | 0.81 | 1.64e-27 | MICROARRAY |
| ***CD72*** | 8 | 1.84 | 7.79e-27 | B-HOT |
| ***MYOZ1*** | 9 | 1.73 | 1.5e-26 | MICROARRAY |
| ***CYLD*** | 10 | 0.65 | 1.5e-26 | MICROARRAY |
| ***TSPOAP1-AS1*** | 11 | 1.15 | 1.72e-26 | NGS |
| ***RCSD1*** | 12 | 1.19 | 3.68e-26 | MICROARRAY |
| ***BANK1*** | 13 | 1.14 | 1.31e-25 | MICROARRAY |
| ***STK17A*** | 14 | 0.88 | 9.41e-25 | MICROARRAY |
| ***STAMBPL1*** | 15 | 1.02 | 2.77e-24 | MICROARRAY |
| ***ST3GAL5*** | 16 | 0.9 | 2.79e-24 | MICROARRAY |
| ***ARHGEF6*** | 17 | 0.87 | 2.79e-24 | MICROARRAY |
| ***LAG3*** | 18 | 2.02 | 3.06e-24 | B-HOT |
| ***WIPF1*** | 19 | 0.89 | 7.73e-24 | MICROARRAY |
| ***LOC374443*** | 20 | 1.09 | 1.38e-23 | NGS |
| ***STK4*** | 21 | 0.71 | 1.38e-23 | MICROARRAY |
| ***CRLF3*** | 22 | 0.87 | 1.47e-23 | MICROARRAY |
| ***RGS10*** | 23 | 1.10 | 2.05e-23 | MICROARRAY |
| ***CD8A*** | 24 | 2.23 | 2.16e-23 | B-HOT |
| ***MIR3142HG*** | 25 | 1.18 | 2.56e-23 | NGS |
| ***FNBP1*** | 26 | 0.57 | 3.03e-23 | MICROARRAY |
| ***MSL3*** | 27 | 0.60 | 3.33e-23 | MICROARRAY |
| ***TRAF5*** | 28 | 0.81 | 5.21e-23 | MICROARRAY |
| ***CD28*** | 29 | 1.88 | 5.25e-23 | B-HOT |
| ***ARPC2*** | 30 | 0.40 | 5.25e-23 | MICROARRAY |

**Supplementary Figure 1: Distribution of the time from transplantation**

The figure shows the time from transplantation (x-axis) in function of the number of biopsies (y-axis).

**Supplementary Figure 2: Gene panels distribution across the antibody-mediated rejection signature**

Density plot of the proportions of transcripts related to the NGS, the B-HOT and the microarray gene panels**.** The NGS-specific proportions are highlighted in red, B-HOT in yellow, and the microarray in blue. X-axis represents the cumulative increasing adjusted p-value-ranked top X genes and the y-axis represents the proportion of each gene panel. B-HOT-related genes are mainly represented in the top genes while the microarray-related genes are highly represented throughout the entire signature and the NGS-specific genes are constantly represented for approximately 10% of the genes.

*Abbreviations: B-HOT: Banff Human Organ Transplant; NGS: next generation sequencing*

**Supplementary Figure 3: Top 30 overall enriched pathways in antibody-mediated rejection**

**

**Supplementary Figure 4: Set of cytokines and cytokine receptors engaged in the antibody-mediation rejection signature**

KEGG pathway visualization for the set of cytokines and cytokine receptors. Transcripts have been colored according to their log_2_ fold change with blue (red) values corresponding to down- (up-)regulated genes, highlighting group of transcripts interconnection by shared arrows. Grey markers correspond to missing markers in the signature. The range of colors has been bounded to [-1,1] to limit the impact of very high/low log_2_ fold changes (min=-1.55, max=2.28).

**
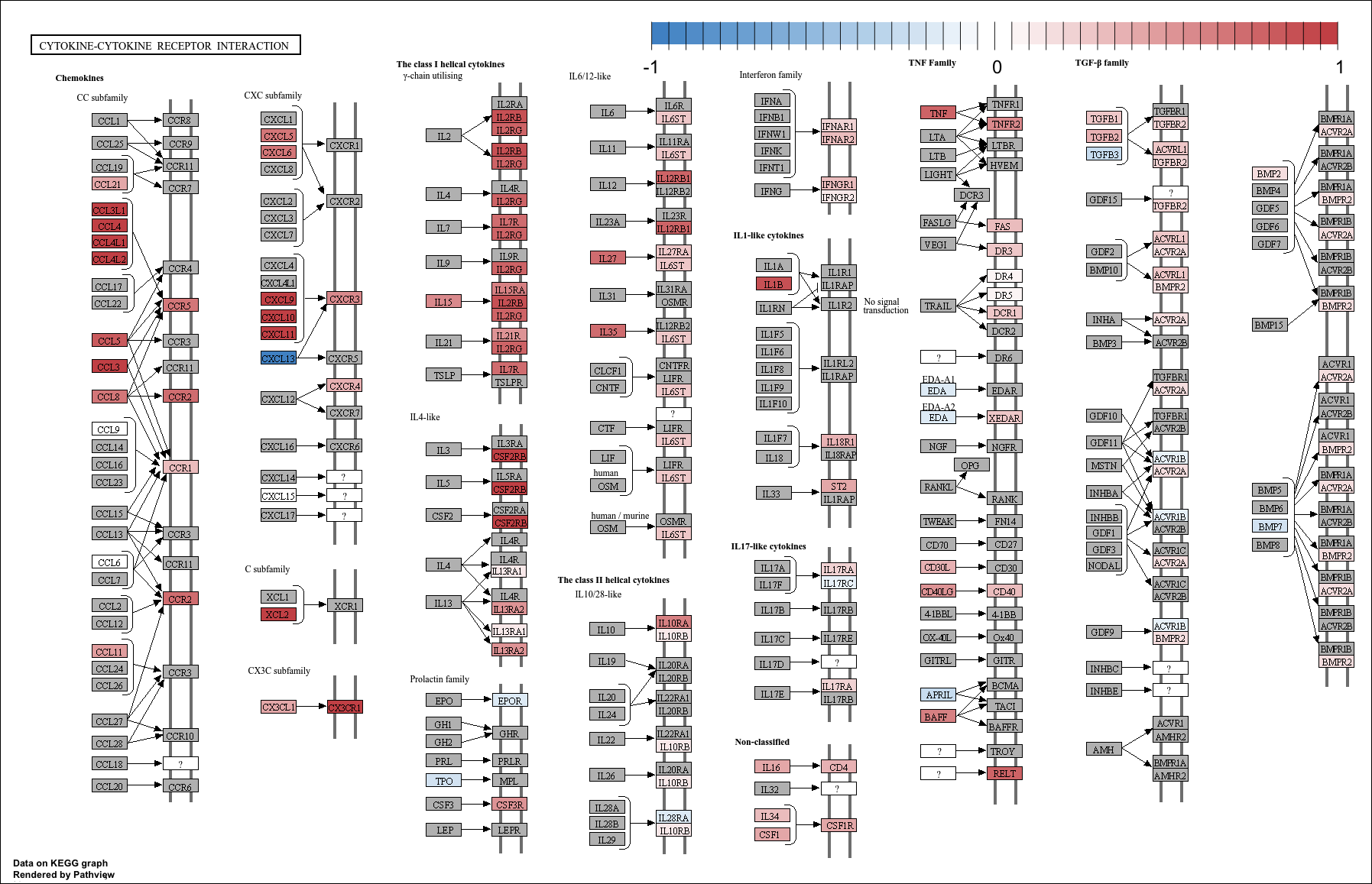
**

**Supplementary Figure 5: Set of cell adhesion molecules engaged in the antibody-mediation rejection signature**

KEGG pathway visualization for the set of cell adhesion molecules. Transcripts have been colored according to their log_2_ fold change with blue (red) values corresponding to down- (up-)regulated genes. Grey markers correspond to missing markers in the signature. The range of colors has been bounded to [-1,1] to limit the impact of very high/low log_2_ fold changes (min=-1.55, max=2.28).

**
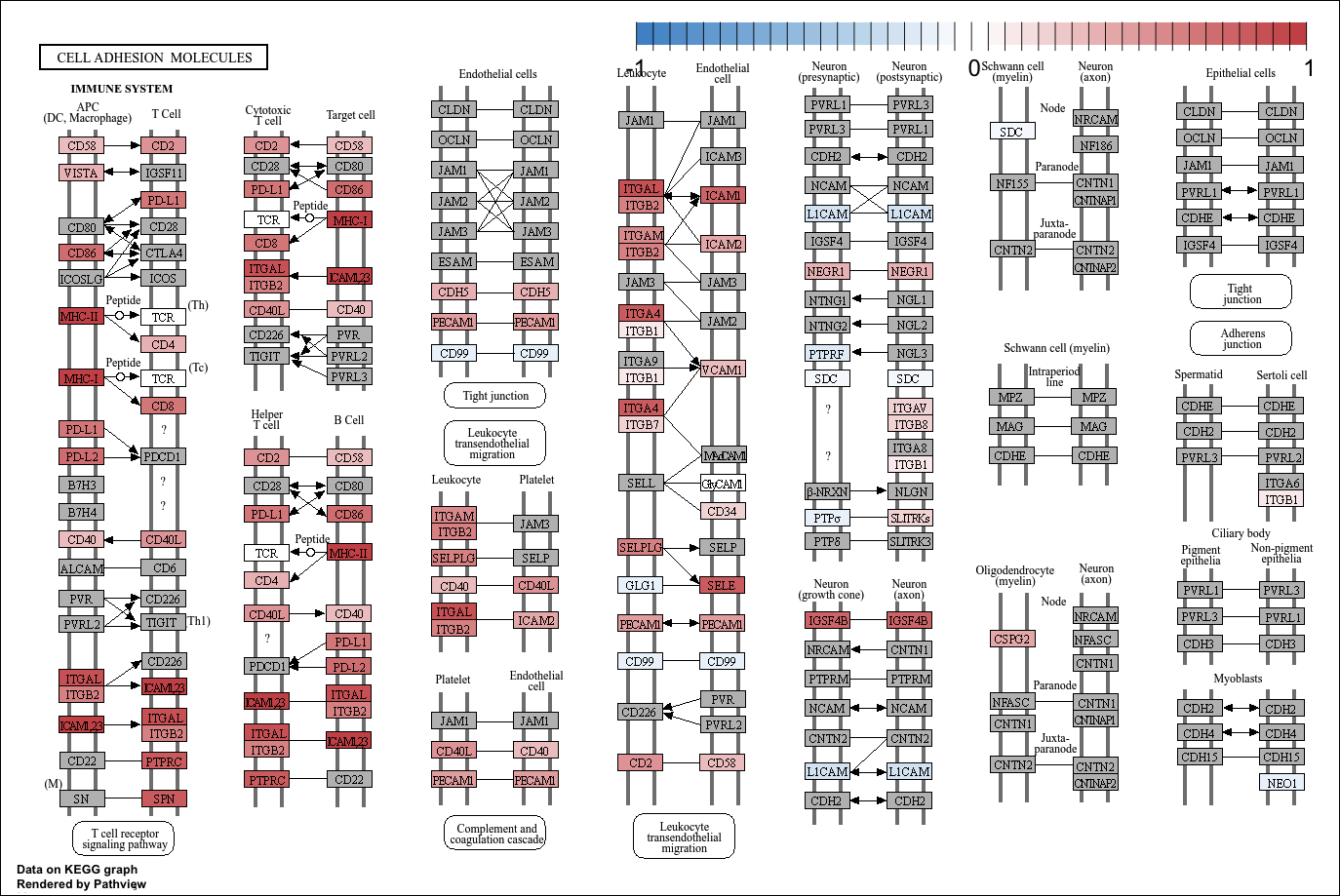
**

**Supplementary Figure 6: Antibody-mediated rejection map of enriched pathways (BHOT-specific up-regulated gene list)**

Map of enriched pathways involved in the antibody-mediated rejection B-HOT-specific up-regulated genes. The interaction map contextualizes the pathophysiological categories inter-relations. Vertices represent pathways, dots color intensity refers to the significance of the category and sized of the dots is in accordance to the number of genes in the signature. Edges symbolize the overlap between two pathways, powered by the shared transcripts. The closer two vertices are and the thicker is the edge connecting them, the wider is the overlap between the two pathways.

**

**Supplementary Figure 7: Antibody-mediated rejection map of enriched pathways (microarray-specific up-regulated gene list)**

Map of enriched pathways involved in the antibody-mediated rejection microarray-specific up-regulated genes. The interaction map contextualizes the pathophysiological categories inter-relations. Vertices represent pathways, dots color intensity refers to the significance of the category and sized of the dots is in accordance to the number of genes in the signature. Edges symbolize the overlap between two pathways, powered by the shared transcripts. The closer two vertices are and the thicker is the edge connecting them, the wider is the overlap between the two pathways.

**

**Supplementary Figure 8: Antibody-mediated rejection map of enriched pathways (NGS-specific up-regulated gene list)**

Map of enriched pathways involved in the antibody-mediated rejection NGS-specific up-regulated genes. The interaction map contextualizes the pathophysiological categories inter-relations. Vertices represent pathways, dots color intensity refers to the significance of the category and sized of the dots is in accordance to the number of genes in the signature. Edges symbolize the overlap between two pathways, powered by the shared transcripts. The closer two vertices are and the thicker is the edge connecting them, the wider is the overlap between the two pathways.

**

**Supplementary Figure 9: Gene panels distribution across the T-cell mediated rejection signature**

Density plot of the proportions of transcripts related to the NGS, the B-HOT and the microarray gene panels**.** The NGS-specific proportions are highlighted in red, B-HOT in yellow, and the microarray in blue. X-axis represents the cumulative increasing adjusted p-value-ranked top X genes and the y-axis represents the proportion of each gene panel.

Abbreviations: BHOT: Banff Human Organ Transplant; NGS: next generation sequencing.

**Supplementary Figure 10: Set of cell adhesion molecules engaged in the T-cell mediation rejection signature**

KEGG pathway visualization for the set of cell adhesion molecules. Transcripts have been colored according to their log_2_ fold change with blue (red) values corresponding to down- (up-)regulated genes. Grey markers correspond to missing markers in the signature. The range of colors has been bounded to [-2,2] to limit the impact of very high/low log_2_ fold changes (min=-1.8, max=3.9).


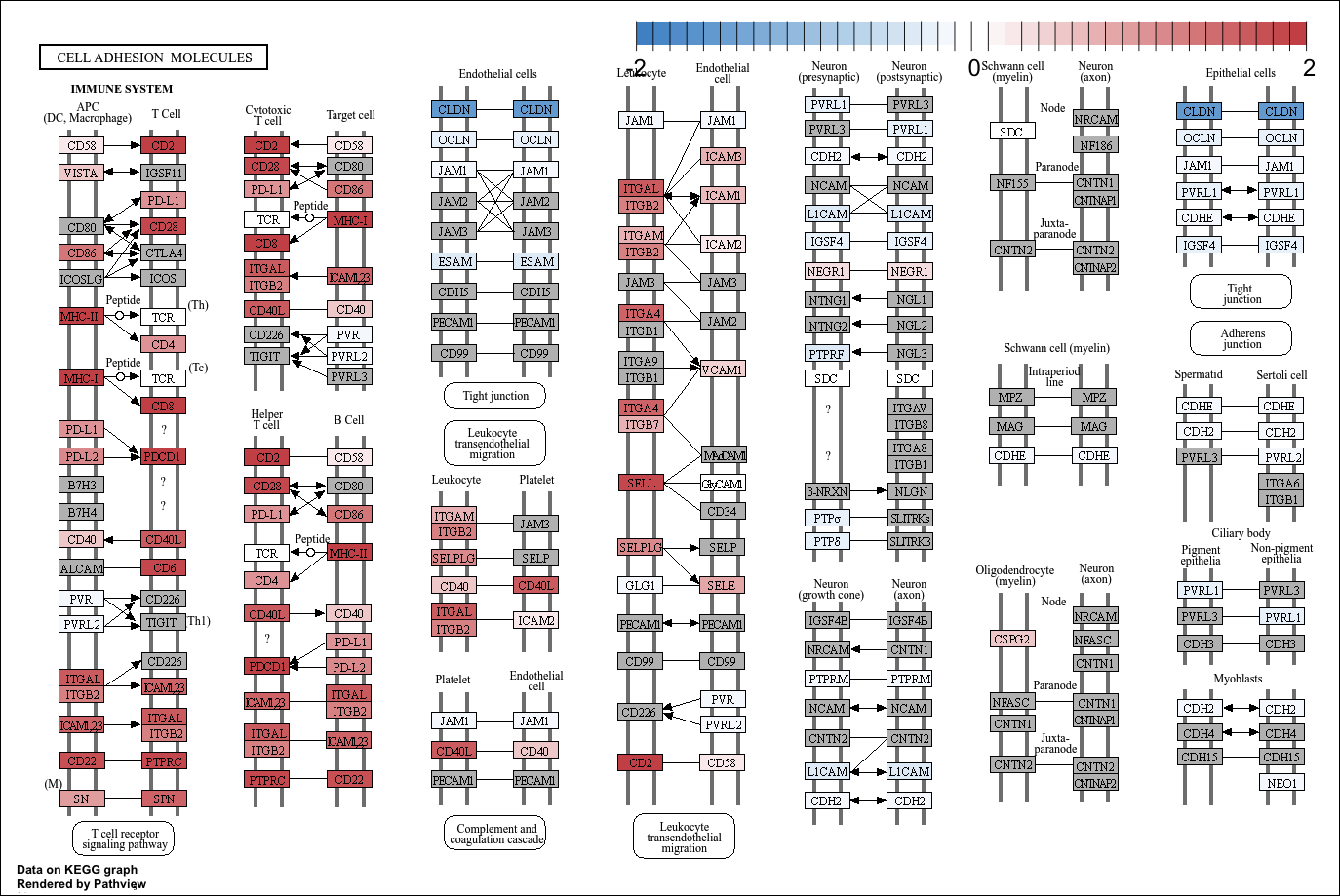


**Supplementary Figure 11: T-cell mediated rejection map of enriched pathways (BHOT-specific up-regulated gene list)**

Map of enriched pathways involved in the antibody-mediated rejection B-HOT-specific up-regulated genes. The interaction map contextualizes the pathophysiological categories inter-relations. Vertices represent pathways, dots color intensity refers to the significance of the category and sized of the dots is in accordance to the number of genes in the signature. Edges symbolize the overlap between two pathways, powered by the shared transcripts. The closer two vertices are and the thicker is the edge connecting them, the wider is the overlap between the two pathways.

**Supplementary Figure 12: T-cell mediated rejection map of enriched pathways (microarray-specific up-regulated gene list)**

Map of enriched pathways involved in the antibody-mediated rejection microarray-specific up-regulated genes. The interaction map contextualizes the pathophysiological categories inter-relations. Vertices represent pathways, dots color intensity refers to the significance of the category and sized of the dots is in accordance to the number of genes in the signature. Edges symbolize the overlap between two pathways, powered by the shared transcripts. The closer two vertices are and the thicker is the edge connecting them, the wider is the overlap between the two pathways.

**References**

1. Maxwell® RSC miRNA Tissue Kit Technical Manual #TM441

2. Illumina® Stranded mRNA Prep Ligation

Document # 1000000124518 v01

3. Andrews S. (2010). FastQC: a quality control tool for high throughput sequence data.

4. Dobin A, Davis CA, Schlesinger F, Drenkow J, Zaleski C, Jha S, et al. STAR: ultrafast universal RNA-seq aligner. Bioinformatics. 2013;29(1):15-21.

5. Picard Toolkit. 2018. Broad Institute, GitHub Repository. <http://broadinstitute.github.io/picard/>

6. Wang L, Wang S, & Li W (2012). RSeQC: quality control of RNA-seq experiments. Bioinformatics(Oxford, England), 28(16), 2184–2185.
